# A Rare Constellation of Triple Cutaneous Malignancies With Sustained Therapeutic Response: Kaposi Sarcoma, Basal Cell Carcinoma, and Cutaneous Squamous Cell Carcinoma in a Single Patient

**DOI:** 10.7759/cureus.94604

**Published:** 2025-10-14

**Authors:** Ivan Bivolarski

**Affiliations:** 1 Medical Oncology, Integrated Oncology Centre Burgas, Burgas, BGR

**Keywords:** basal cell carcinoma diagnosis, kaposi sarcoma paclitaxel, multiple skin cancers, scc-squamous cell carcinoma, therapeutic response

## Abstract

The simultaneous occurrence of three distinct cutaneous malignancies - Kaposi sarcoma (KS), basal cell carcinoma (BCC), and cutaneous squamous cell carcinoma (cSCC) - in a single patient is exceptionally uncommon. We report the case of an 83-year-old male with cardiovascular comorbidities who was diagnosed with KS following histopathological confirmation (CD34⁺, ERG⁺, HHV-8⁺) and had a prior history of excised BCC and cSCC of the skin between 2020 and 2022. Over the course of his illness, he underwent multiple surgical resections, axillary lymph node dissection, systemic chemotherapy with paclitaxel, and immunotherapy. Despite eventual disease progression, he achieved sustained therapeutic benefit for more than 18 months, with clinical stabilization of lesions and meaningful palliation.

This rare constellation underscores the diagnostic and therapeutic challenges of managing multiple primary skin malignancies in an immunosenescent patient. The coexistence of KS with keratinocyte carcinomas may reflect overlapping carcinogenic mechanisms, including viral oncogenesis (human herpesvirus-8 (HHV-8)), ultraviolet exposure, and immune dysregulation. Vigilant lesion-by-lesion biopsy, histopathological confirmation, and individualized multimodal management can achieve durable disease control and improved quality of life, even in elderly patients with complex dermatologic oncologic presentations.

## Introduction

Kaposi sarcoma (KS) is an angioproliferative neoplasm linked to human herpesvirus-8 (HHV-8) infection and typically arises in the context of immune dysregulation or advanced age [1,2]. In contrast, basal cell carcinoma (BCC) and cutaneous squamous cell carcinoma (cSCC) are the two most common non-melanoma skin cancers worldwide, strongly associated with cumulative ultraviolet (UV) exposure, photodamage, and field cancerization [3,4].

While BCC and cSCC often coexist in the same patient due to overlapping etiological factors, the occurrence of KS together with both keratinocyte carcinomas is exceptionally rare. Such constellations pose diagnostic challenges, as morphologically distinct lesions may be misattributed to a single pathology. Therapeutically, management requires a lesion-specific approach that balances oncologic control with age-related comorbidities.

This article reports the case of an 83-year-old man with biopsy-proven KS, BCC, and cSCC who achieved more than 18 months of clinical stability under multimodal therapy. The case underscores the importance of systematic lesion-by-lesion evaluation, histopathological confirmation, and individualized treatment planning in complex cutaneous oncology.

## Case presentation

An 83-year-old male with a history of cardiovascular comorbidities (hypertensive heart disease, chronic ischemic heart disease, and prior myocardial infarction in 2022) presented with a unique oncologic trajectory involving three distinct cutaneous malignancies: basal cell carcinoma (BCC), cutaneous squamous cell carcinoma (cSCC), and Kaposi sarcoma (KS).

The oncologic history began in October 2020, when a biopsy of a facial lesion revealed keratinizing basosquamous carcinoma. In March 2022, a biopsy of an axillary lymph node confirmed squamous cell carcinoma, initially suspected to represent inflammatory disease. In April 2023, surgical excision of a cutaneous lesion again demonstrated squamous cell carcinoma.

In February 2022, the patient was admitted with left axillary lymphadenopathy. Partial dissection was performed, but histology revealed lipogranuloma rather than metastatic disease. Subsequent PET/CT detected metabolically active nodes on the contralateral side, and additional biopsies confirmed Kaposi sarcoma, with immunohistochemistry positive for CD34 and ERG.

Based on these findings, systemic paclitaxel chemotherapy was initiated. The patient received six cycles of first-line paclitaxel, followed by a short course of immunotherapy with cemiplimab (three cycles), and subsequently four cycles of second-line paclitaxel upon progression, achieving several months of clinical stability.

In February 2024, new ulceroinfiltrative lesions on the left auricle and right chest wall were excised, and histopathology confirmed moderately differentiated cSCC (G2) with clear margins. In April 2024, paclitaxel was reinitiated (six cycles through September 2024). HIV 1/2 testing at that time was negative. Later that month, a biopsy of another auricular lesion confirmed well-differentiated cSCC (G1).

**Figure 1:**
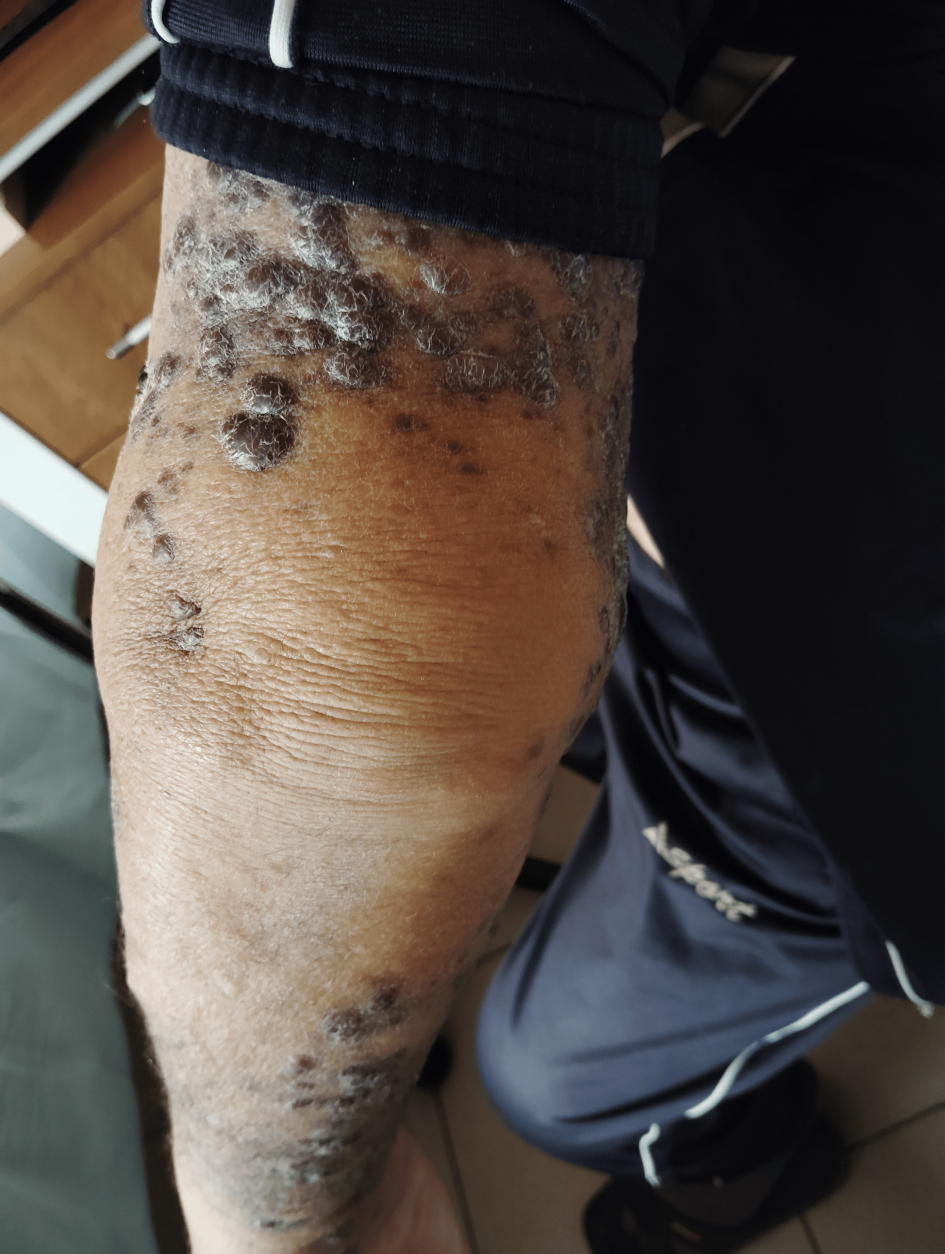
Cutaneous Kaposi sarcoma lesions on the left arm and chest before systemic therapy The lesions appear as multiple violaceous nodules and plaques, some confluent, with associated skin thickening and mild edema. At this stage, the disease was histologically confirmed by biopsy with immunohistochemistry (CD34⁺, ERG⁺, HHV-8⁺). These findings illustrate the extent of cutaneous involvement that prompted initiation of systemic paclitaxel chemotherapy.

Before initiation of first-line systemic therapy, the patient exhibited multiple violaceous nodules and plaques on the left arm and chest, consistent with cutaneous Kaposi sarcoma (Figure 1).

**Figure 2:**
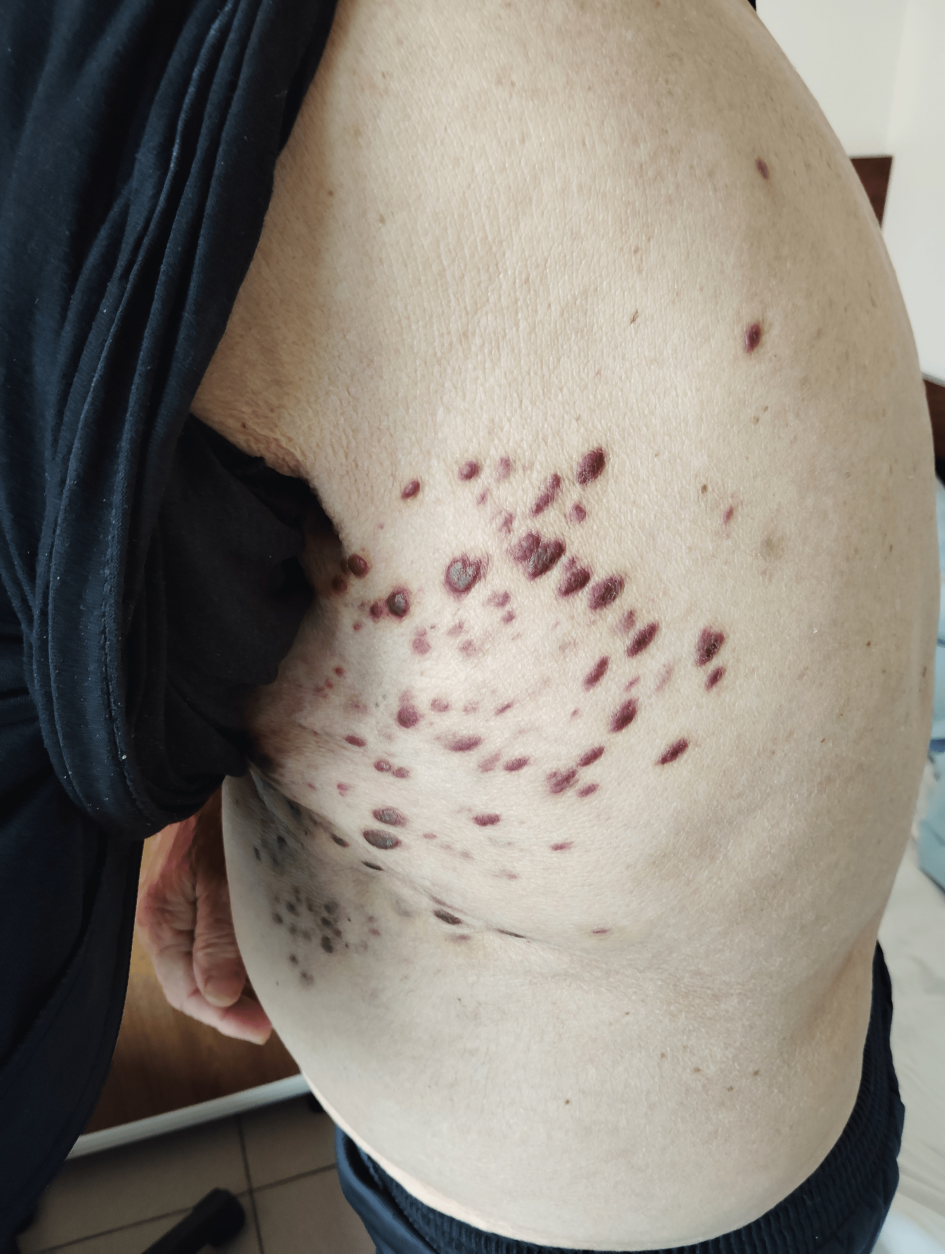
In addition to the lesions on the extremities, the patient also developed multiple violaceous nodules and plaques on the left hemithorax.

The lesions were confluent in places, with associated skin thickening and mild edema (Figure 2). Histological confirmation with immunohistochemistry (CD34⁺, ERG⁺, HHV-8⁺) established the diagnosis. The extent and progression of these lesions prompted the initiation of systemic paclitaxel chemotherapy.

**Figure 3:**
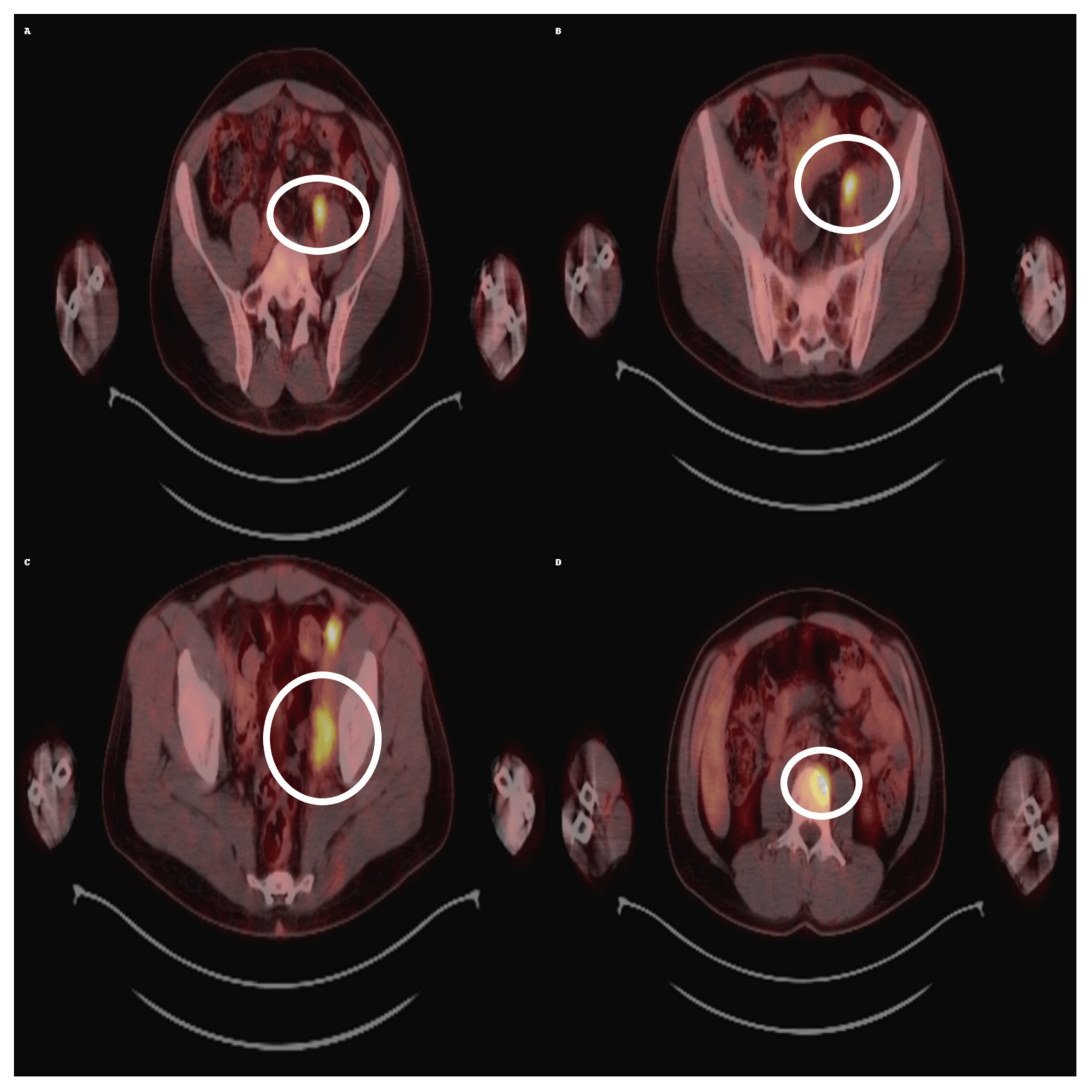
Baseline PET/CT scan showing hypermetabolic lesions in the pelvis and spleen. Axial fused PET/CT images (A-D) demonstrate increased uptake consistent with Kaposi sarcoma involvement. The regions of interest are indicated by the white ellipses, with the maximum standardized uptake value (SUVmax) ranging between 3.9 and 6.4 at this stage.

Sequential PET/CT imaging documented disease dynamics: in March 2024, metabolically active lesions were present in the forearm and spleen (maximum standardized uptake value (SUVmax) 6.38) (Figure 3).

**Figure 4:**
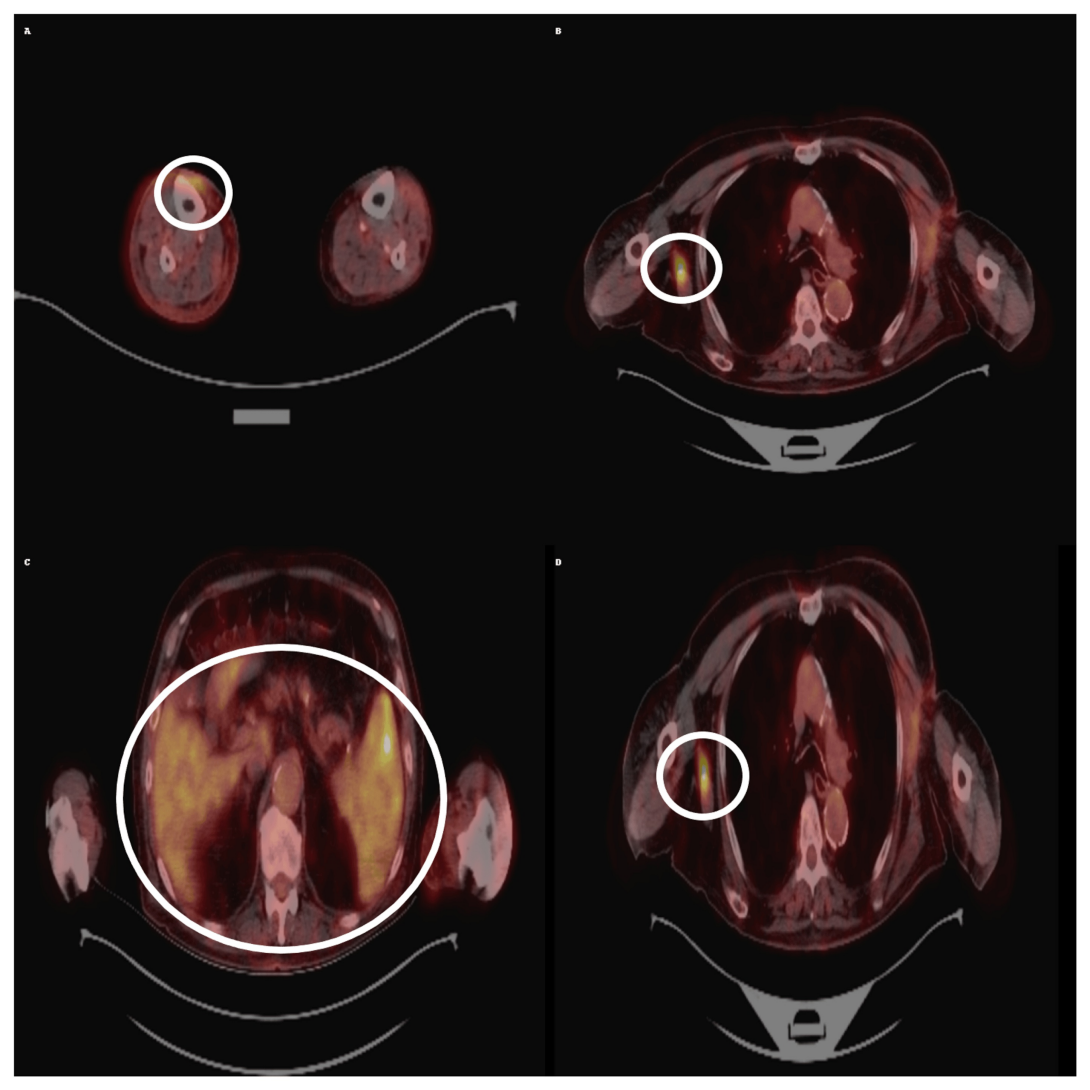
PET/CT scan in November 2024 showing disease progression with new hypermetabolic lesions. Axial fused PET/CT images (A-D) demonstrate new hypermetabolic lesions in the extremities, mediastinum, and spleen. The regions of interest are indicated by white ellipses, with SUVmax increasing up to 15.5, consistent with aggressive Kaposi sarcoma activity.

In August 2024, a near-complete metabolic response was observed, with resolution of forearm and splenic uptake, decreased axillary uptake (SUVmax 2.54), and only residual activity in a left auricular lesion. However, in November 2024, progression occurred with new hypermetabolic lesions in the mediastinum, axilla, and spleen (SUVmax up to 15.5) (Figure 4).

**Figure 5:**
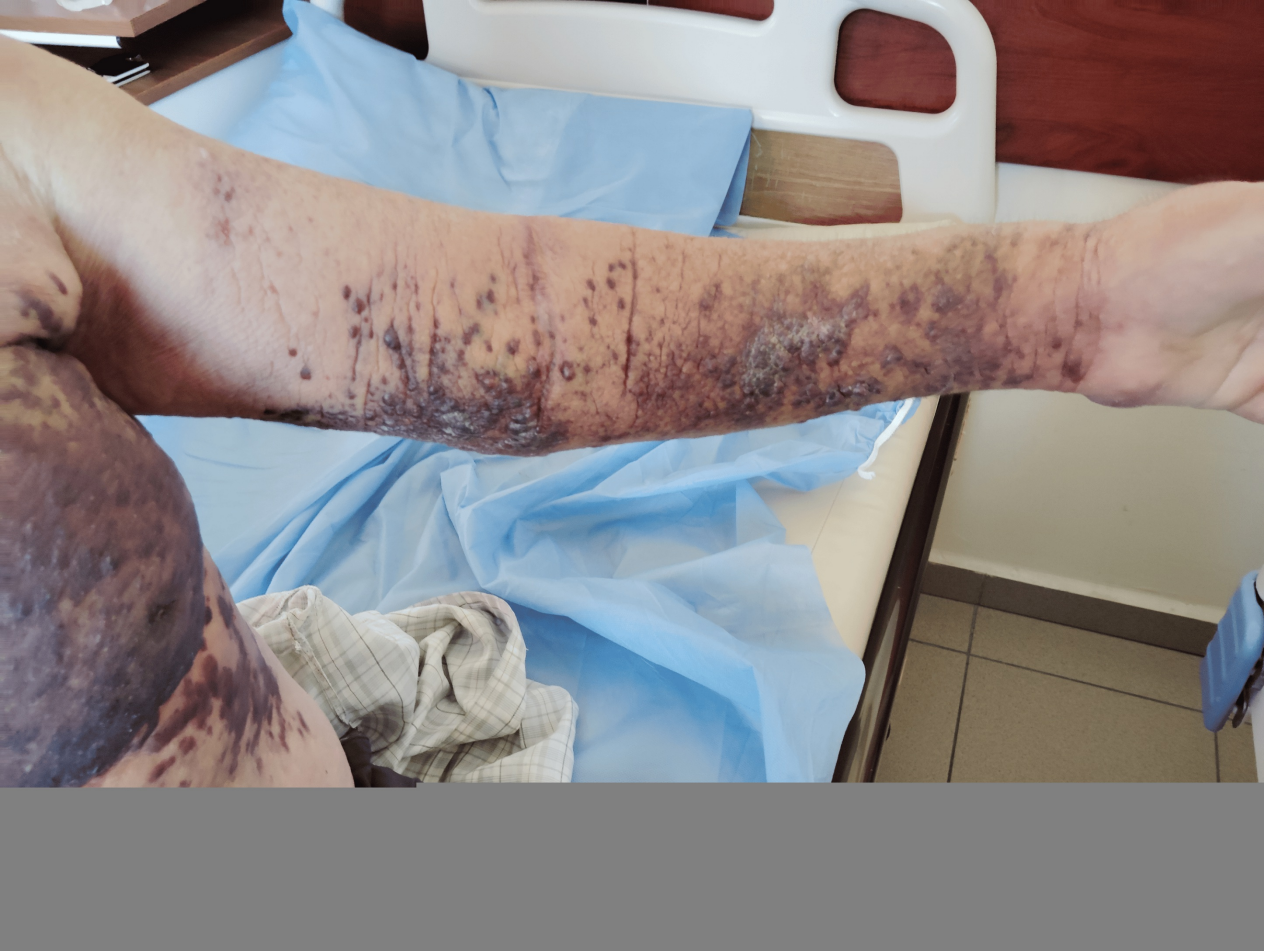
Advanced Kaposi sarcoma lesions involving the left arm. Extensive violaceous plaques and nodules with confluent infiltration and edema reflect advanced disease progression.

**Figure 6:**
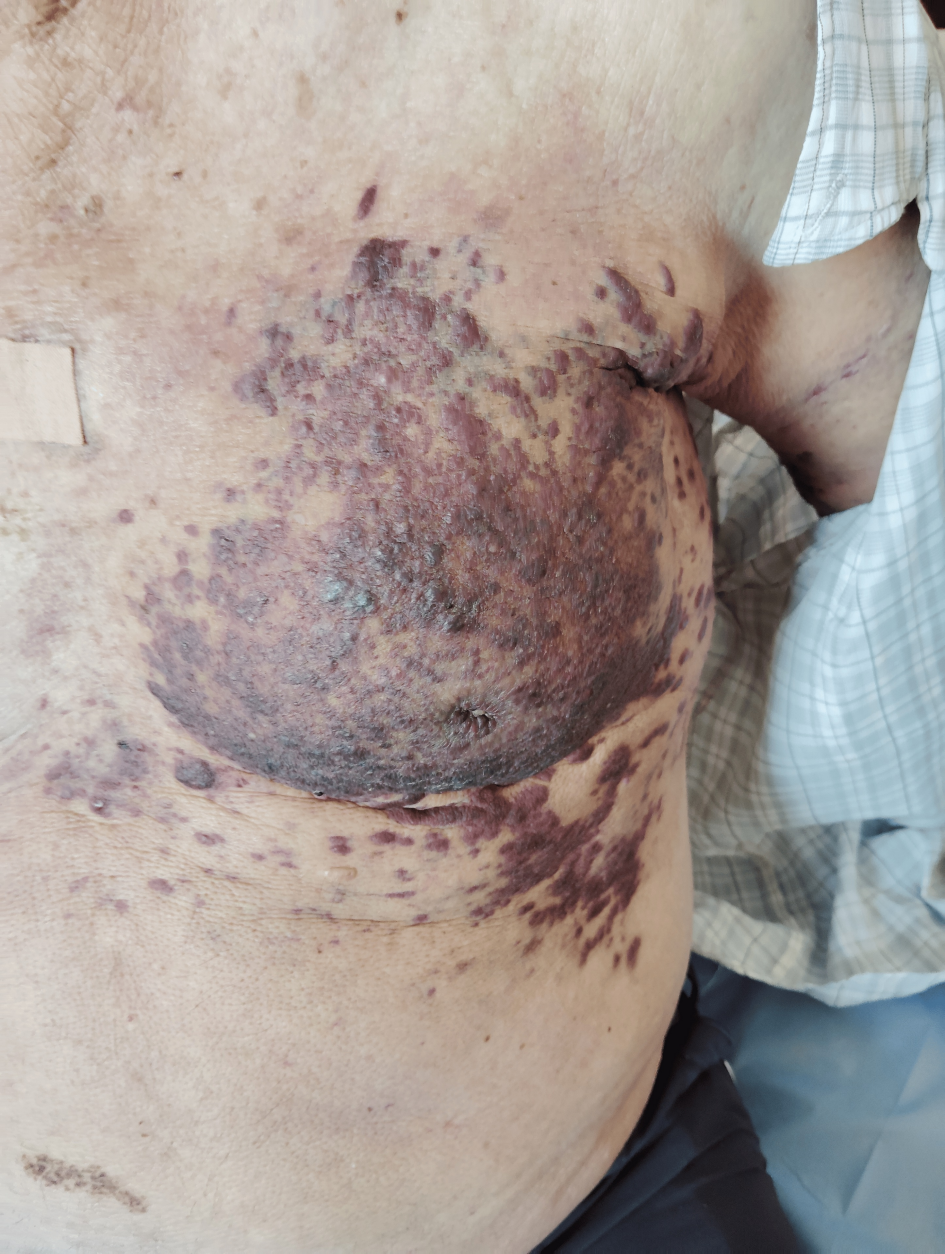
Kaposi sarcoma progression on the anterior chest wall after treatment failure. Extensive, confluent violaceous and dark-brown plaques demonstrate marked infiltration and skin thickening, contrasting with the earlier scattered nodular distribution. These findings reflect advanced cutaneous progression consistent with the patient’s deteriorating clinical status.

Meanwhile, in October 2024, histopathology confirmed recurrent squamous cell carcinoma in the facial region, and the patient was started on cemiplimab immunotherapy. By November 2024, however, KS lesions clinically progressed with enlarging nodules on the face, prompting discontinuation of immunotherapy and resumption of chemotherapy (Figures 5, 6).

**Figure 7:**
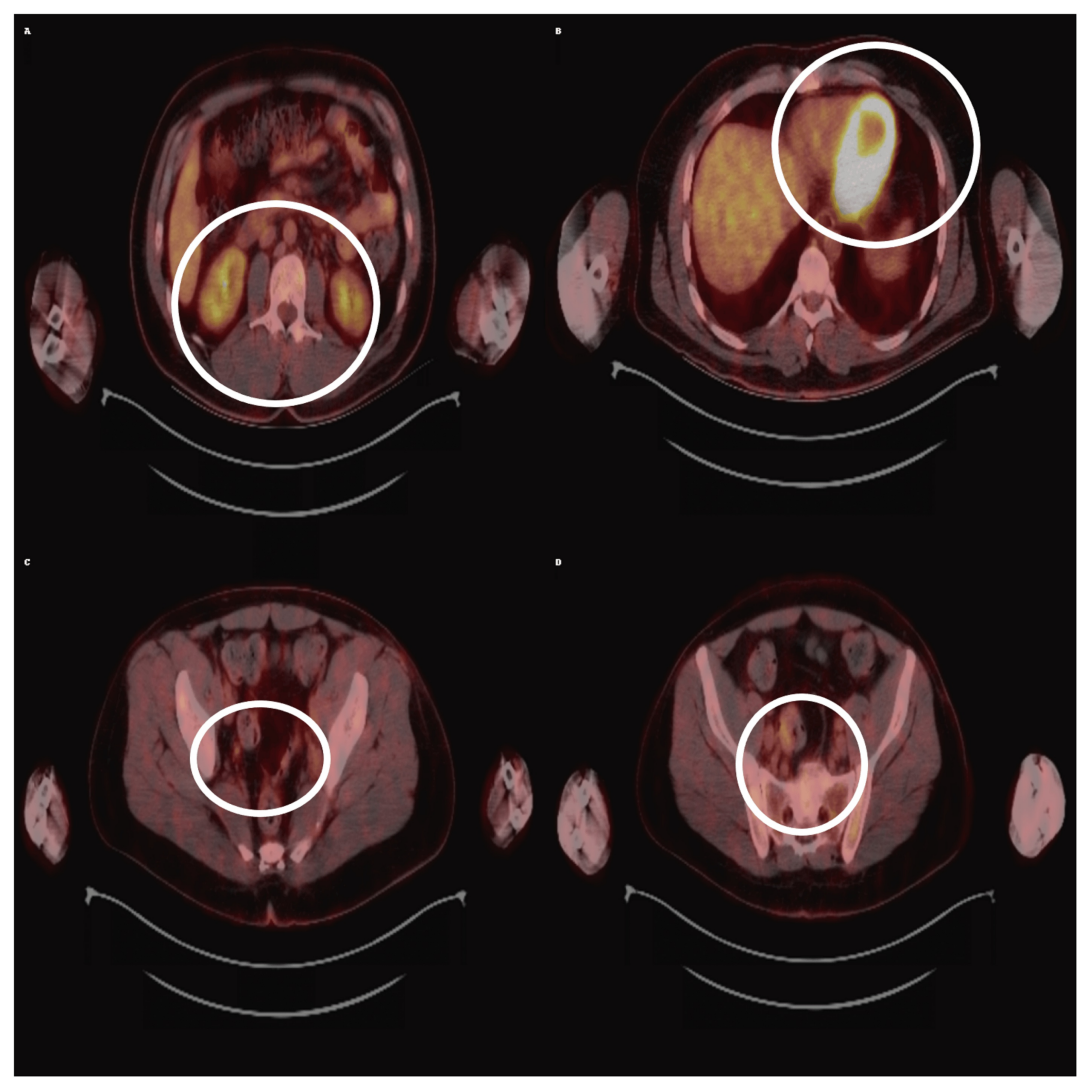
PET/CT scan in April 2025 showing partial metabolic response after systemic therapy. Axial fused PET/CT images (A-D) demonstrate a marked reduction in lesion uptake compared to November 2024, with standardized uptake value (SUVmax) decreasing to 1.78. Mild splenic uptake persisted, as indicated by the white ellipses.

In January 2025, paclitaxel rechallenge was initiated. A follow-up PET/CT in April 2025 showed partial metabolic response, with regression of several lesions and reduction in SUVmax to 1.78 (Figure 7).

By June 2025, the patient’s condition deteriorated. He exhibited disseminated violaceous nodular and plaque-like lesions across all extremities with mild edema, as well as infiltrative lesions on the face, including the right eyelid and left auricle. He was admitted for palliative care on June 27, 2025 and died on July 6, 2025 after nine days of hospitalization.

Despite advanced age, comorbidities, and the coexistence of three distinct skin malignancies, the patient maintained over 18 months of disease control with multimodal therapy, which highlights the importance of individualized oncologic management.

## Discussion

The coexistence of multiple primary skin cancers is relatively common in elderly patients, largely due to cumulative UV exposure, field cancerization, and age-related immune decline [2]. Basal cell carcinoma (BCC) and cutaneous squamous cell carcinoma (cSCC) are frequently observed in the same individual and are collectively referred to as non-melanoma skin cancers (NMSC) [5,6]. In contrast, Kaposi sarcoma (KS) is a vascular neoplasm driven by human herpesvirus-8 (HHV-8), typically arising in the context of immunosenescence, HIV infection, or iatrogenic immunosuppression [1,7,8].

The synchronous or metachronous occurrence of KS together with both BCC and cSCC is exceedingly rare, with only isolated cases described in the literature [9,10]. The present case illustrates the diagnostic and therapeutic challenges of such a constellation. Clinically, violaceous plaques and nodules of KS may mimic vascular or inflammatory dermatoses, whereas hyperkeratotic or ulceroinfiltrative cSCC lesions demand prompt biopsy and surgical excision. A lesion-by-lesion diagnostic approach proved critical in enabling accurate identification of each distinct pathology.

Several overlapping mechanisms may explain the development of multiple cutaneous malignancies in this patient. These include chronic UV exposure [5], viral oncogenesis (HHV-8) [1,2], age-related immune dysfunction, and possibly a shared proangiogenic microenvironment [9,11]. Additionally, the patient’s cardiovascular comorbidities and advanced age may have impaired immune surveillance, further predisposing to tumorigenesis.

From a therapeutic perspective, the case demonstrates the value of individualized, multimodal management. The patient underwent surgical excision for localized cSCC lesions, received multiple lines of paclitaxel chemotherapy for KS, and was briefly treated with cemiplimab immunotherapy for recurrent cSCC. Although disease progression eventually occurred, he achieved more than 18 months of meaningful disease control and symptom palliation. These findings suggest that even elderly, comorbid patients can derive sustained benefit when treatments are carefully sequenced [12].

Learning points

The case presents the following lessons: (1) Biopsy each morphologically distinct lesion in patients with a history of multiple skin cancers to avoid diagnostic anchoring; (2) Recognize that the coexistence of viral-driven and UV-induced malignancies is possible, particularly in immunosenescent hosts; and (3) Apply multimodal, lesion-specific treatment to achieve sustained disease control and improve quality of life, even in advanced age.

## Conclusions

An exceptionally rare case of triple cutaneous malignancies - Kaposi sarcoma, basal cell carcinoma, and cutaneous squamous cell carcinoma - arose sequentially in an elderly patient. This constellation highlights the importance of maintaining a high index of suspicion and performing lesion-specific biopsies whenever multiple morphologies are observed.

Despite advanced age, cardiovascular comorbidities, and a complex oncologic history, the patient achieved more than 18 months of sustained clinical benefit through a multimodal strategy incorporating surgery, chemotherapy, and immunotherapy. Carefully tailored, lesion-specific management can provide meaningful disease control and quality-of-life improvement, even in patients with multiple primary skin cancers.
